# Surveillance for Eurasian-origin and intercontinental reassortant highly pathogenic influenza A viruses in Alaska, spring and summer 2015

**DOI:** 10.1186/s12985-016-0511-9

**Published:** 2016-03-31

**Authors:** Andrew M. Ramey, John M. Pearce, Andrew B. Reeves, Rebecca L. Poulson, Jennifer Dobson, Brian Lefferts, Kyle Spragens, David E. Stallknecht

**Affiliations:** U.S. Geological Survey, Alaska Science Center, 4210 University Drive, Anchorage, AK 99508 USA; Southeastern Cooperative Wildlife Disease Study, Department of Population Health, College of Veterinary Medicine, 589 D. W. Brooks Drive, University of Georgia, Athens, GA 30602 USA; Yukon-Kuskokwim Health Corporation, 900 Chief Eddie Hoffman Highway, Bethel, AK 99559 USA; U.S. Geological Survey, Western Ecological Research Center, San Francisco Bay Estuary, 505 Azuar Drive, Vallejo, CA 94592 USA; U.S. Fish and Wildlife Service, Yukon Delta National Wildlife Refuge, 807 Chief Eddie Hoffman Highway, Bethel, AK 99559 USA

**Keywords:** Alaska, H5N1, H5N2, H5N8, Highly pathogenic, Influenza, Migratory bird, Yukon-Kuskokwim Delta

## Abstract

**Background:**

Eurasian-origin and intercontinental reassortant highly pathogenic (HP) influenza A viruses (IAVs) were first detected in North America in wild, captive, and domestic birds during November–December 2014. Detections of HP viruses in wild birds in the contiguous United States and southern Canadian provinces continued into winter and spring of 2015 raising concerns that migratory birds could potentially disperse viruses to more northerly breeding areas where they could be maintained to eventually seed future poultry outbreaks.

**Results:**

We sampled 1,129 wild birds on the Yukon-Kuskokwim Delta, Alaska, one of the largest breeding areas for waterfowl in North America, during spring and summer of 2015 to test for Eurasian lineage and intercontinental reassortant HP H5 IAVs and potential progeny viruses. We did not detect HP IAVs in our sample collection from western Alaska; however, we isolated five low pathogenic (LP) viruses. Four isolates were of the H6N1 (*n =* 2), H6N2, and H9N2 combined subtypes whereas the fifth isolate was a mixed infection that included H3 and N7 gene segments. Genetic characterization of these five LP IAVs isolated from cackling (*Branta hutchinsii*; *n =* 2) and greater white-fronted geese (*Anser albifrons*; *n =* 3), revealed three viral gene segments sharing high nucleotide identity with HP H5 viruses recently detected in North America. Additionally, one of the five isolates was comprised of multiple Eurasian lineage gene segments.

**Conclusions:**

Our results did not provide direct evidence for circulation of HP IAVs in the Yukon-Kuskokwim Delta region of Alaska during spring and summer of 2015. Prevalence and genetic characteristics of LP IAVs during the sampling period are concordant with previous findings of relatively low viral prevalence in geese during spring, non-detection of IAVs in geese during summer, and evidence for intercontinental exchange of viruses in western Alaska.

**Electronic supplementary material:**

The online version of this article (doi:10.1186/s12985-016-0511-9) contains supplementary material, which is available to authorized users.

## Background

In November–December 2014, highly pathogenic (HP) H5 influenza A viruses (IAVs) of the A/goose/Guandong/1/1996 (Gs/Gd) lineage were first detected in wild, captive, and domestic birds in North America [1–4]. HP IAVs of the H5N8 subtype in North America were genetically similar at all eight gene segments to viruses causing recent poultry outbreaks in South Korea and Japan [1, 2], whereas those of the H5N2 and H5N1 subtypes were comprised of gene segments of North American and Eurasian ancestral lineages [1, 3, 4]. The timing and route by which Gs/Gd lineage HP H5 IAVs were introduced to North America are still unclear; however, several studies have suggested wild migrating birds to be a potential mechanism of introduction from Asia [2, 5, 6].

Genetic and ecological data from the Republic of Korea provide evidence for: (1) an association between the detection of Gs/Gd lineage HP H5 IAVs and the presence of both wild and domestic waterfowl, (2) few detections of HP IAVs during summer months when migratory waterfowl are largely absent from the region, and (3) evolution of HP IAVs between wintering seasons for migratory birds in a currently unknown reservoir population [5]. This scenario is parsimonious with viral maintenance at northerly breeding areas for migratory birds; however, other explanations such as non-detection of Gs/Gd lineage IAVs in resident domestic and wild birds cannot be ruled out. Similarly, detections of HP IAVs in wild birds and domestic poultry occurred repeatedly in North America during winter and spring of 2015, but tapered off by summer, raising the possibility that migratory birds could disperse viruses to northerly breeding areas where they may be maintained to eventually seed future poultry outbreaks as birds migrate back to wintering grounds. Alternatively, HP IAVs recently detected in North America may not be maintained in the wild bird reservoir and therefore may not be found at northern latitude breeding areas despite surveillance efforts.

To assess the evidence for migratory birds dispersing HP H5 IAVs from lower latitude wintering areas to northern breeding grounds, we sampled wild birds during spring and summer at one of the largest breeding grounds in North America, the Yukon-Kuskokwim Delta, Alaska. We sampled hunter-harvested birds from ten remote villages in the spring and live-captured birds during summer to test for infection with IAVs, specifically screening for Gs/Gd lineage HP H5 viruses. Although sampling in remote regions of Alaska such as the Yukon-Kuskokwim Delta provides logistical challenges for surveillance, information derived from this effort provides an opportunity to gather evidence for or against the hypothesis that wild birds maintain Gs/Gd HP H5 IAVs through spring migration with relatively little potential for confounding associated with anthropogenic activities such as poultry production.

## Methods

**Fig. 1:**
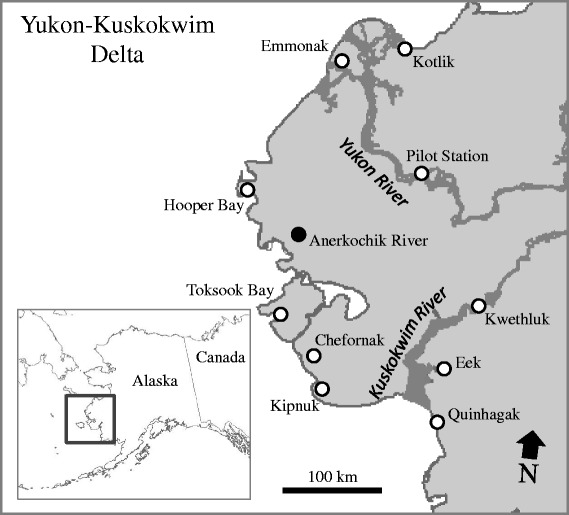
Map of the Yukon-Kuskowim Delta, Alaska and approximate locations for wild bird surveillance sampling for Eurasian lineage and intercontinental reassortant highly pathogenic influenza A viruses during spring (open circles) and summer (darkened circle) 2015

Hunter-harvested birds were sampled for IAVs at ten villages within the Yukon-Kuskokwim Delta, Alaska (Fig. 1) during May 2015 as part of the subsistence season. This is a traditional harvest period for native people living in this region during which migratory birds are harvested for personal use. Subsistence hunters brought intact harvested birds for viral sampling to health clinics located in each village: Chefornak, Eek, Emmonak, Hooper Bay, Kipnuk, Kotlik, Kwethluk, Pilot Station, Quinhagak, and Toksook Bay (Fig. 1). At sampling stations for each village, swab samples for viral screening were collected from each bird by applying a polyester-tipped applicator to each the oropharynx/trachea and the cloaca and swabbing. Paired samples were then combined in a single cryovial with 2 ml of chilled viral transport media supplemented with antibiotics. Samples were then placed directly in dewars charged with liquid nitrogen and transferred to -80 ° C freezers until testing.

In summer, cackling geese (*Branta hutchinsii*) were sampled at remote sites near the Anerkochik River (Fig. 1) during 16–19 July as part of banding efforts conducted by the Yukon Delta National Wildlife Refuge. Geese were captured during the flightless molting period in drives using lead fences to guide birds into a capture pen with assistance from a helicopter and 6-person ground crew to encourage directionality of bird movements (United States Department of the Interior federal bird banding permit #09811; USFWS-Region 7 IACUC #2015-009). After capture, and application of a metal tarsal band, paired oropharyngeal and cloacal swabs were collected as previously explained for hunter-harvested birds (United States Department of the Interior federal bird banding permit #20022; USGS Alaska Science Center ACUC #2015-13). Samples were kept cool/frozen upon collection until transfer to -80 ° C freezers within approximately one week of collection.

In the laboratory, all paired swab samples were screened using rRT-PCR targeting gene products for the IAV matrix gene [7] and the Gs/Gd lineage HP H5 hemagglutinin gene segment [8]. All spring samples yielding Ct values ≤ 41 for the IAV matrix gene and all summer samples (regardless of Ct value for the IAV matrix gene) were inoculated into specific pathogen-free embryonated chicken eggs for virus isolation [9]. Amnio-allantoic fluids were collected after 120 h, and tested via hemagglutination assay. RNAs extracted from hemagglutination assay positive amnio-allantoic fluids were screened for the IAV matrix gene by rRT-PCR [10] and resultant isolates were genomically sequenced [11] (GenBank; accession #’s: KU310452–KU310492). Pairwise nucleotide distance comparisons were made between sequences for all gene segments for viruses isolated as part of this study and those for the following representative Eurasian lineage and intercontinental reassortant HP IAVs detected in North America using MEGA6 [12]: A/American green-winged teal/WA/19570/2014 (H5N1), A/northern pintail/WA/40964/2014 (H5N2), and A/gyrfalcon/WA/41009-6/2014 (H5N8). To infer the continental lineage of each internal gene segment of isolates recovered from the Yukon-Kuskokwim Delta, maximum-likelihood phylogenies were reconstructed for each gene segment in MEGA6 [12] using 1,000 bootstrap replicates and representative reference sequences for viruses originating from Eurasia and North America. As phylogenetic analysis may have limited utility for inferring continental lineages of surface glycoproteins, we used the standard NCBI nucleotide Basic Local Alignment Search Tool (BLAST) to gain inference on closely related viral strains (BLAST conducted 4 December 2015).

## Results

We did not detect HP H5 IAVs from 1,129 birds sampled on the Yukon-Kuskokwim Delta, Alaska, during spring and summer 2015. However, we did detect five low pathogenic (LP) IAVs from samples collected from cackling and greater white-fronted geese (*Anser albifrons*) during spring (Table 1). Samples were collected from at least 36 different species of wild birds (Table 1); however, the sample collection was biased by taxonomic order (waterfowl 1,014/1,129; 90 %) and season (spring 1,014/1,129; 90 %). The overall virus isolation rate for our sample collection was 0.4 % (5/1,129). Species specific virus isolation rates were 1.0 % (2/200) and 1.0 % (3/298) for cackling and greater white-fronted geese in spring, respectively, and 0 % for all other species in spring and cackling geese in summer.

**Table 1:** Summary of wild bird surveillance sampling for Eurasian lineage and intercontinental reassortant highly pathogenic influenza A viruses on the Yukon-Kuskokwim Delta, Alaska during spring and summer 2015

Spring surveillance (7–29 May 2015)			
Species	*n =*	rRT-PCR +^a^	VI +^b^
American green-winged teal (*Anas crecca*)	4	0	-
American wigeon (*Anas americana*)	1	0	-
Arctic tern (*Sterna paradisaea*)	1	1	0
Bar-tailed godwit (*Limosa lapponica*)	14	1	0
Black brant (*Branta bernicla nigricans*)	41	3	0
Black scoter (*Melanitta americana*)	83	4	0
Black turnstone (*Arenaria melanocephala*)	3	0	-
Cackling goose (*Branta hutchinsii*)	200	7	2
Common goldeneye (*Bucephala clangula*)	2	0	-
Common merganser (*Mergus merganser*)	1	0	-
Dunlin (*Calidris alpina*)	9	1	0
Emperor goose (*Chen canagica*)	19	4	0
Gadwall (*Anas strepera*)	1	0	-
Greater scaup (*Aythya marila*)	11	2	0
Greater white-fronted goose (*Anser albifrons*)	298	39	3
Lapland longspur (*Calcarius lapponicus*)	1	0	-
Lesser scaup (*Aythya affinis*)	62	7	0
Long-billed dowitcher (*Limnodromus scolopaceus*)	3	1	0
Long-tailed duck (*Clangula hyemalis*)	6	1	0
Mallard (*Anas platyrhynchos*)	12	0	-
Mew gull (*Larus canus*)	2	0	-
Northern pintail (*Anas acuta*)	15	1	0
Northern shoveler (*Anas clypeata*)	19	1	0
Pacific loon (*Gavia pacifica*)	1	1	0
Pectoral sandpiper (*Calidris melanotos*)	16	1	0
Red-necked phalarope (*Phalaropus lobatus*)	23	3	0
Rock sandpiper (*Calidris ptilocnemis*)	1	0	-
Sabine’s gull (*Xema sabini*)	1	1	0
Sandhill crane (*Grus canadensis*)	31	2	0
Sharp-tailed sandpiper (*Calidris acuminata*)^c^	3	0	-
Snow Goose (*Chen caerulescens*)	62	5	0
Surf Scoter (*Melanitta perspicillata*)	5	1	0
Tundra swan (*Cygnus columbianus*)	41	3	0
White-winged scoter (*Melanitta deglandi*)	16	1	0
Willow ptarmigan (*Lagopus lagopus*)	3	0	-
Wilson’s snipe (*Gallinago delicata*)	1	0	-
Unknown species (Class Aves)	2	1	0
spring total	1014	92	5
Summer surveillance (16–19 July 2015)			
species	*n =*	rRT-PCR +^a^	VI +^b^
Cackling goose	115	2	0

^a^based on *Ct* value < 41

^b^only spring samples with *Ct* values < 41 subjected to VI; all summer samples subjected to VI

^c^rare report for this species at this location during spring; field identification was not verified by trained ornithologist

Four isolates from cackling and greater white-fronted geese were of the H6N1 (*n =* 2), H6N2, and H9N2 combined subtypes. A fifth isolate was the result of a mixed infection that included H3 and N7 gene segments. Three isolates originated from samples collected in Pilot Station (A/cackling goose/Alaska/UGAI15-3902/2015 [H6N1], A/greater white-fronted goose/Alaska/UGAI15-3903/2015 [H6N1], and A/greater white-fronted goose/Alaska/UGAI15-3913/2015 [H6N2]) whereas one isolate originated from each Hooper Bay (A/cackling goose/Alaska/UGAI15-3075/2015 [H9N2]) and Toksook Bay (A/greater white-fronted goose/Alaska/UGAI15-4167/2015[H3N7]). Shared nucleotide sequence identity between LP IAV strains isolated from Yukon-Kuskokwim Delta geese and representative strains of HP IAV isolated in North America in 2014–2015, was generally less than 99 % with few exceptions (Additional file 1: Tables S1–S8). One of the two NP sequences (variant #2) for A/greater white-fronted goose/Alaska/UGAI15-4167/2015 (mixed) shared 99 % identity with A/northern pintail/WA/40964/2014 (H5N2) (Additional file 1: Table S5). Additionally, the N1 NA gene segments for A/greater white-fronted goose/Alaska/UGAI15-3903/2015 (H6N1) and A/cackling goose/Alaska/UGAI15-3902/2015 (H6N1) shared 99 % identity with A/American green-winged teal/WA/19570/2014 (H5N1) (Additional file 1: Table S6).

Nucleotide sequences for internal gene segments of IAVs isolated from cackling and greater white-fronted geese were of North American lineages for four isolates (Additional file 2: Figures S1–S6). In contrast, A/greater white-fronted goose/Alaska/UGAI15-4167/2015 (mixed) appeared to include gene segments of both North American and Eurasian lineages. Sequences for the PB2, NP (variant #1), M, and NS (variant #1) gene segments of A/greater white-fronted goose/Alaska/UGAI15-4167/2015 (mixed) were of Eurasian lineage (Additional file 2: Figures S1, S4–S6) whereas sequences for the PB1, NP (variant #2), and NS (variant #2) gene segment appeared to be of North American lineage (Additional file 2: Figures S2, S4, and S6). NCBI BLAST provided similar results as compared to phylogenetic analyses with top hits for HA and NA gene segments for four of five isolates from geese providing evidence for shared nucleotide identity of 96–99 % with isolates derived from dabbling ducks sampled in North America, including the American green-winged teal sampled in Washington and infected with a Gs/Gd lineage HP H5N1 IAV (Additional file 1: Table S9). In contrast, top BLAST hits for sequences for HA and NA gene segments from A/greater white-fronted goose/Alaska/UGAI15-4167/2015 (mixed) indicated 97 % shared nucleotide identity with IAVs isolated from wild birds sampled in Eurasia (Additional file 1: Table S9).

## Discussion

We did not detect any direct evidence for the circulation of HP H5 IAVs in wild birds sampled on the Yukon-Kuskokwim Delta, Alaska, during spring or summer of 2015. Probable explanations include that HP H5 IAVs were not present in the region or that prevalence of such viruses were sufficiently low in local wild waterfowl so as to preclude detection during sampling periods. Other explanations include that HP H5 IAVs were circulating in waterfowl species poorly represented in our sample collection or non-waterfowl species that were not sampled during our collection efforts.

Sampling of hunter-harvested birds from a relatively large number of villages spread across the region likely provides representative information regarding IAV prevalence for the Yukon-Kuskokwim Delta for the sampling period, particularly for species for which we were able to obtain larger sample sizes. Unlike baited trap sites which may increase viral transmission among animals using capture locations [13], hunter-harvested birds are more likely to be independent samples and therefore reflective of natural infection prevalence in a study system. Despite a considerable sampling effort on the Yukon-Kuskokwim Delta during spring and summer, few LP IAVs were recovered from our sample collection. However, the rate at which we isolated viruses in two species of geese sampled in spring was comparable to reported estimated viral prevalence for four species of geese in this region during spring 2006–2010 using rRT-PCR (1.78 %) [14]. The lack of isolation of viruses from other avian species or cackling geese during summer is also consistent with low recovery of viruses from dabbling ducks sampled in this region during spring [15] and low estimates for viral prevalence in geese during summer (0 %) [14].

Genetic characterization of five LP IAVs provided only limited evidence for circulation of similar genetic diversity as previously found in Gs/Gd lineage HP IAVs in North America; however, this diversity could also be maintained in the absence of HP IAVs in this region. Although it is possible that gene segments found to be highly similar to HP intercontinental reassortant IAVs is a function of recent presence of such viruses in the Yukon-Kuskokwim Delta region, it is likely that North American lineage gene segments sharing recent common ancestry with those detected in HP intercontinental reassortant viruses circulated in the Pacific Flyway prior to the introduction or evolution of these IAVs in North America. As such, gene segments sharing high similarity with intercontinental reassortant HP IAVs may continue to circulate independent of outbreaks of HP influenza in North America. Similar to this investigation, gene segments sharing high nucleotide identity with intercontinental reassortant HP H5N1 and H5N2 IAVs were detected in migratory waterfowl sampled at nearby Izembek National Wildlife Refuge in autumn of 2014 [6].

Four combined subtypes for LP IAVs detected in cackling and greater white-fronted geese were not previously reported in an investigation genetically characterizing 90 LP IAVs isolated from waterfowl sampled on the Yukon-Kuskokwim Delta between 2006 and 2009 [15]. Additional sampling of geese for IAVs during spring is therefore necessary for understanding seasonal subtype diversity in this region. The finding of four North American lineage IAV isolates from geese sampled on the Yukon-Kuskokwim Delta is not unexpected and is likely a function of migratory tendencies of many geese migrating to the Yukon-Kuskokwim Delta from wintering areas of the Pacific and Central Flyways of North America. However, the identification of numerous Eurasian lineage gene segments in one of the five isolates we recovered supports earlier conclusions that migratory connectivity of birds between the Yukon-Kuskokwim Delta and East Asia also influences IAV dynamics in western Alaska. Ongoing surveillance in western Alaska at locations such as the Yukon-Kuskokwim Delta and Izembek National Wildlife Refuge will continue to provide data on temporal variations in influenza prevalence and diversity as well as the relative frequency of viral exchange between East Asia and North America via Beringia.

## Conclusion

Research and surveillance efforts for IAVs conducted on the Yukon-Kuskokwim Delta, Alaska, during 2015 do not provide any direct evidence for the circulation of Gs/Gd lineage HP H5 IAVs in this region. Possible reasons for lack of detection are numerous. Our results suggest that on-going and additional research and surveillance strategies, such as refined epidemiological modeling, targeting wintering areas of migratory birds [16], and sampling of avian and non-avian taxa in regions with high concentrations of poultry [17], are necessary to understand the distribution and maintenance of Gs/Gd lineage IAVs in North America.

## Additional files

Additional file 1: Nucleotide identity among PB2 (**Table S1**), PB1 (**Table S2**), PA (**Table S3**), HA (**Table S4**), NP (**Table S5**), NA (**Table S6**), M (**Table S7**), and NS (**Table S8**) gene segments of influenza A virus isolates derived from geese sampled at the Yukon-Kuskokwim Delta, Alaska 2015 and and highly pathogenic clade Gs/Gd lineage H5 influenza A virus isolates detected in North America, 2014. Table S9 reports top blast results for hemagglutinin (HA) and neuramindase (NA) gene segments of influenza A virus isolates derived from geese sampled at the Yukon-Kuskokwim Delta, Alaska 2015. (XLSX 31 kb) Additional file 2: Maximum likelihood phylogenies depicting inferred relationship among nucleotide sequences for the PB2 (**Figure S1**), PB1 (**Figure S2**), PA (**Figure S3**), NP (**Figure S4**), M (**Figure S5**), and NS (**Figure S6**) gene segments of influenza A isolates derived from wild geese sampled on the Yukon-Kuskokwim Delta, Alaska during spring 2015 (bold) and others representing continental lineages of influenza A viruses recently detected in North America and Eurasia. Bootstrap support values > 70 for nodes are shown. (PDF 230 kb)

## References

[1] Ip HS, Torchetti MK, Crespo R, Kohrs P, DeBruyn P, Mansfield KG, Baszler T, Badcoe L, Bodenstein B, Shearn-Boschler V, Killian ML, Pedersen JC, Hines N, Gidlewski T, DeLiberto T, Sleeman JM (2015). Novel Eurasian highly pathogenic avian influenza A H5 viruses in wild birds, Washington, USA, 2014. Emerg Infect Dis.

[2] Lee D-H, Torchetti MK, Winker K, Ip HS, Song C-S, Swayne DE (2015). Intercontinental spread of Asian-origin H5N8 to North America through Beringia by migratory birds. J Virol.

[3] Pasick J, Berhane Y, Joseph T, Bowes V, Hisanaga T, Handel K, Alexandersen S (2015). Reassortant highly pathogenic influenza A H5N2 virus containing gene segments related to Eurasian H5N8 in British Columbia, Canada, 2014. Sci Rep.

[4] Torchetti MK, Killian ML, Dusek RJ, Pedersen JC, Hines N, Bodenstein B, White CL, Ip HS (2015). Novel H5 clade 2.3.4.4 reassortant (H5N1) virus from a green-winged teal in Washington, USA. Genome Announc.

[5] Hill SC, Lee Y-J, Song B-M, Kang H-M, Lee E-K, Hanna A, Gilbert M, Brown IH, Prybus OG (2015). Wild waterfowl migration and domestic duck density shape the epidemiology of highly pathogenic H5N8 influenza in the Republic of Korea. Infect Genet Evol.

[6] Ramey AM, Reeves AB, TeSlaa JL, Nashold S, Donnelly T, Bahl J, Hall JS. Evidence for common ancestry among viruses isolated from wild birds in Beringia and highly pathogenic intercontinental reassortant H5N1 and H5N2 influenza A viruses. 2016;40:176-185. 10.1016/j.meegid.2016.02.035 26944444

[7] Spackman E, Senne DA, Myers TJ, Bulaga LL, Garber LP, Perdue ML, Lohman K, Daum LT, Suarez DL (2002). Development of a real-time reverse transcriptase PCR assay for type A influenza virus and the avian H5 and H7 hemagglutinin subtypes. J Clin Microbiol.

[8] U.S. Department of Agriculture. Epidemiologic and other analyses of HPAI-affected poultry flocks: September 9, 2015 Report. 2015; https://www.aphis.usda.gov/animal_health/animal_dis_spec/poultry/downloads/Epidemiologic-Analysis-Sept-2015.pdf. Accessed 17 December 2015.

[9] Stallknecht DE, Shane SM, Zwank PJ, Senne DA, Kearney MT (1990). Avian influenza viruses from migratory and resident ducks of coastal Louisiana. Avian Dis.

[10] Fouchier RAM, Bestebroer TM, Herfst S, Van Der Kemp L, Rimmelzwaan GF (2000). Detection of influenza A viruses from different species by PCR amplification of conserved sequences in the matrix gene. J Clin Microbiol.

[11] Ramey AM, Reeves AB, Sonsthagen SA, TeSlaa JL, Nashold S, Donnelly T, Casler B, Hall JS (2015). Dispersal of H9N2 influenza A viruses between East Asia and North America by wild birds. Virology.

[12] Tamura K, Stecher G, Peterson D, Filipski A (2013). Kumar S MEGA6: Molecular Evolutionary Genetics Analysis version 6.0. Mol Biol Evol.

[13] Soos C, Parmley EJ, McAloney K, Pollard B, Jenkins E, Kibenge F, Leighton FA (2012). Bait trapping linked to higher avian influenza virus detection in wild ducks. J Wildl Dis.

[14] Ely CR, Hall JS, Schmutz JA, Pearce JM, Terenzi J (2013). Evidence that Life History Characteristics of Wild Birds Influence Infection and Exposure to Influenza A Viruses. PLoS One.

[15] Reeves AB, Pearce JM, Ramey AM, Ely CR, Schmutz JA, Flint PL, Derksen DV, Ip HS, Trust KA (2013). Genomic analysis of avian influenza viruses from waterfowl in western Alaska. USA J Wildl Dis.

[16] U.S. Department of Agriculture. Surveillance plan for highly pathogenic avian influenza in waterfowl in the United States. 2015b; http: //www.aphis.usda.gov/animal_health/downloads/animal_diseases/ai/2015-hpai-surveillance-plan.pdf. Accessed 21 August 2015.

[17] Flint PL, Franson JC, Pearce JM, Derksen DV (2015). Wild bird surveillance for highly pathogenic avian influenza H5 in North America. Virol J.

