# Supplementary figures and images for: Surveillance for Eurasian-origin and intercontinental reassortant highly pathogenic influenza A viruses in Alaska, spring and summer 2015
Source: Virol J. 2016 Mar 31;13:55. doi: 10.1186/s12985-016-0511-9 (PMC4815243; doi:10.1186/s12985-016-0511-9)

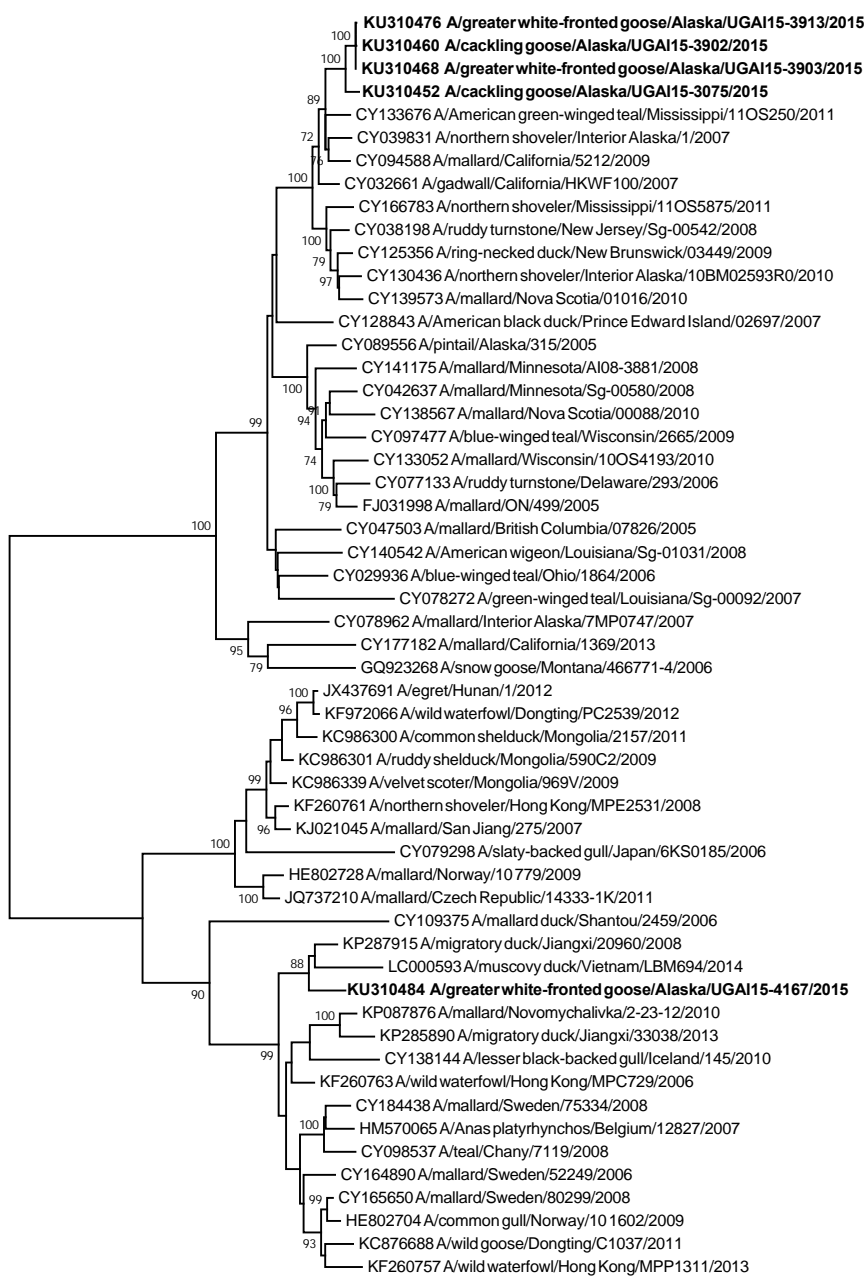

0.05

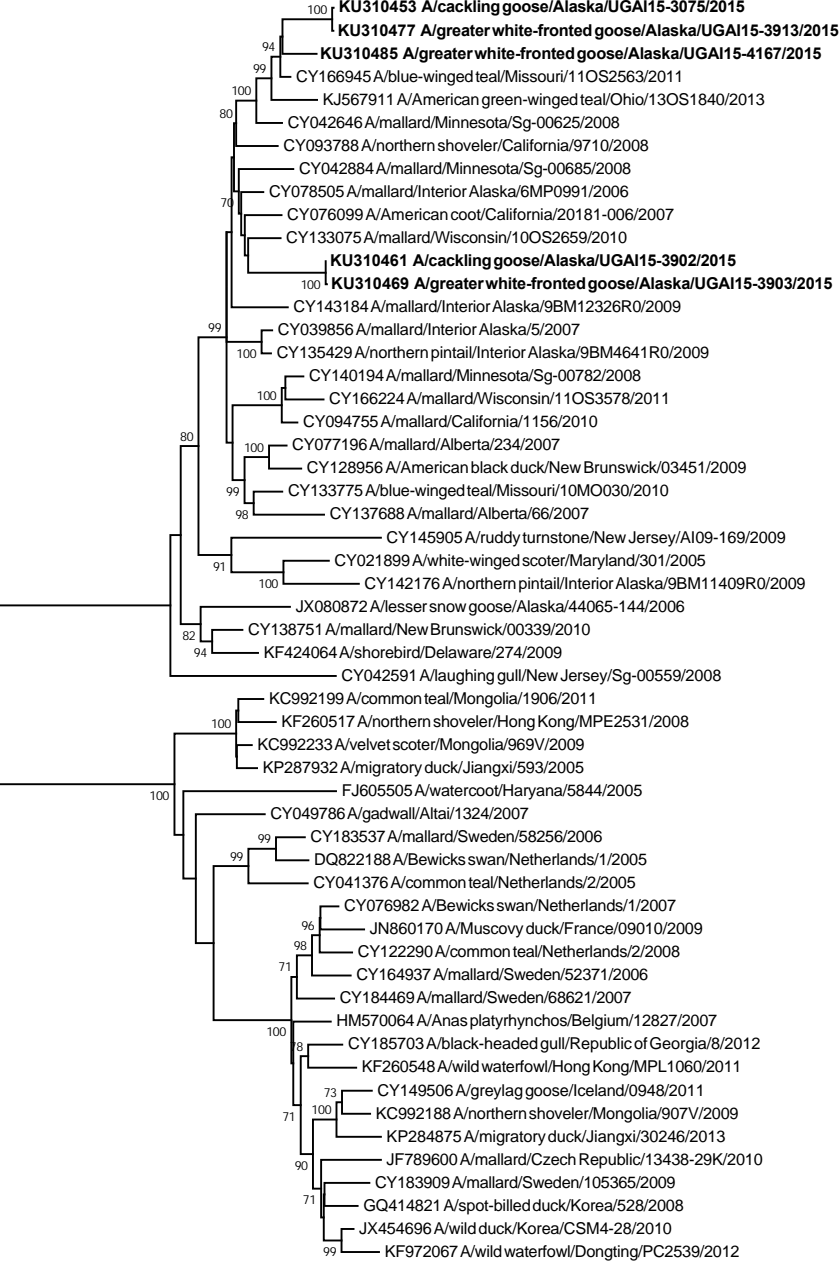

0.02

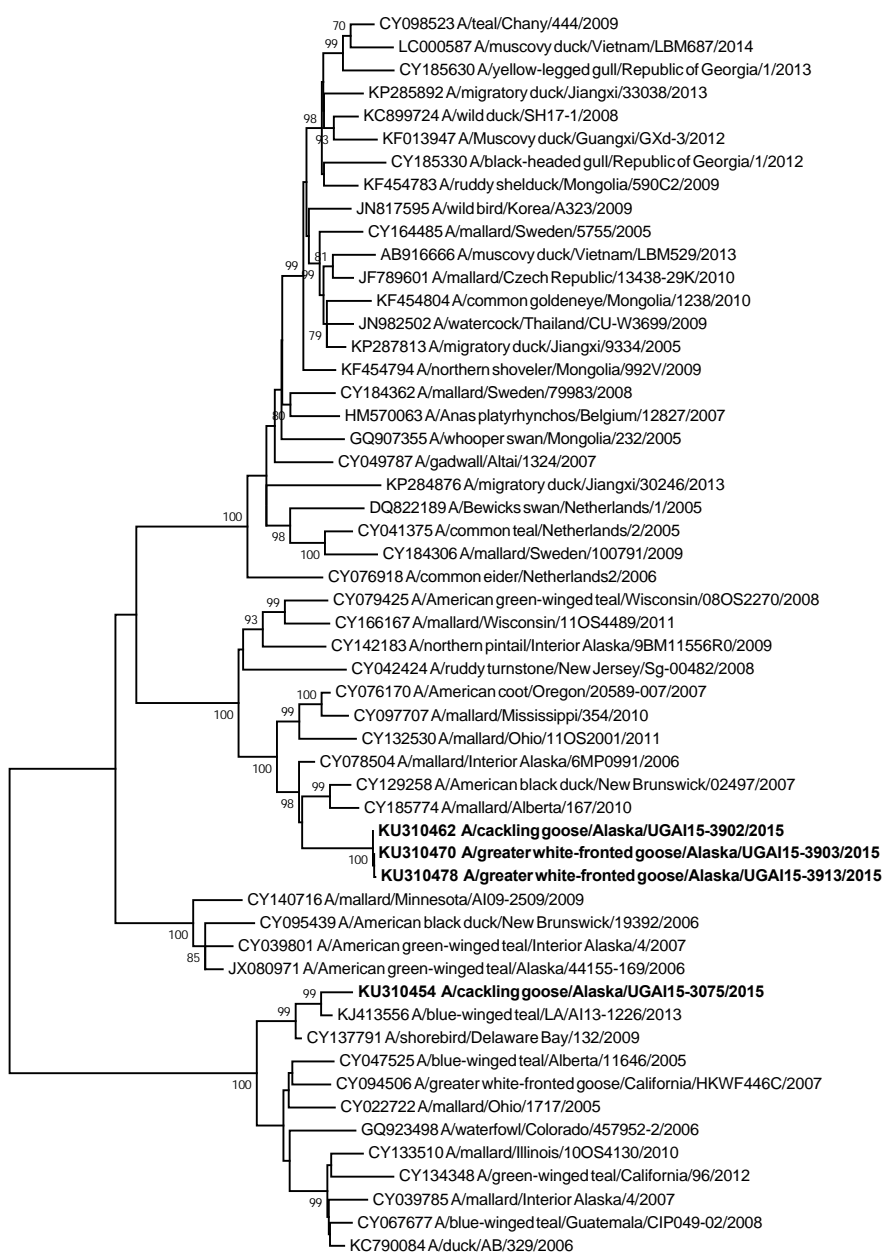

0.02

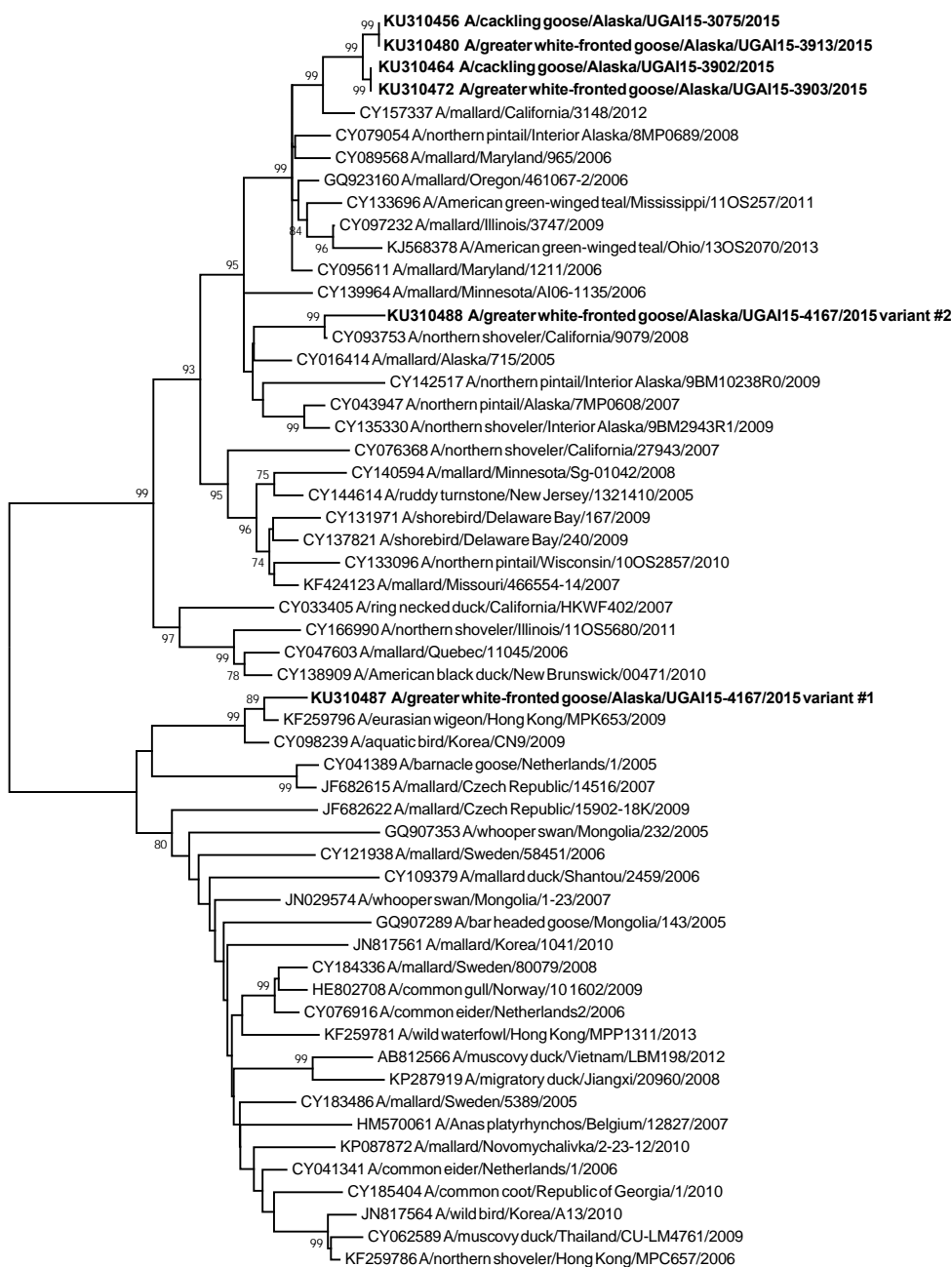

0.02

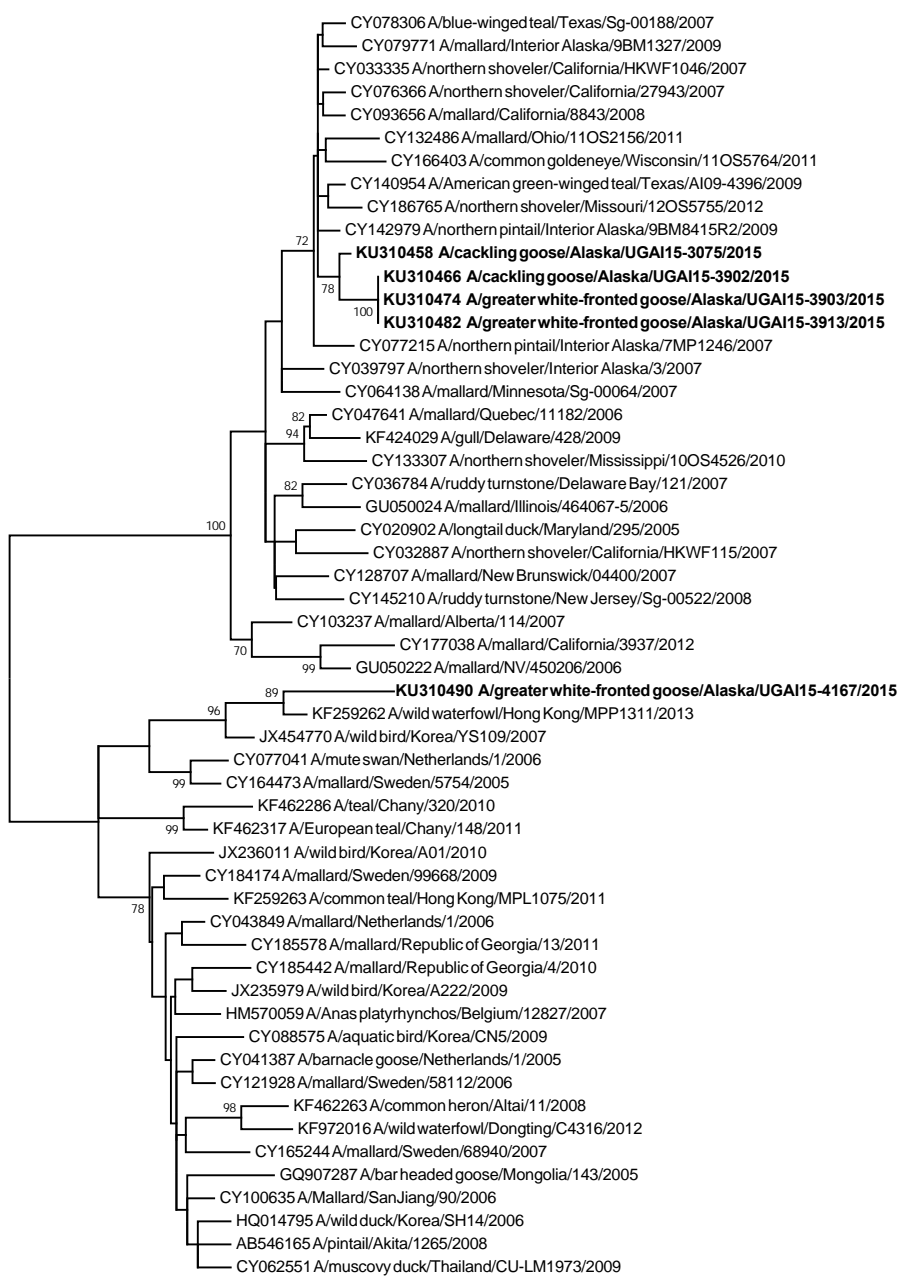

0.01

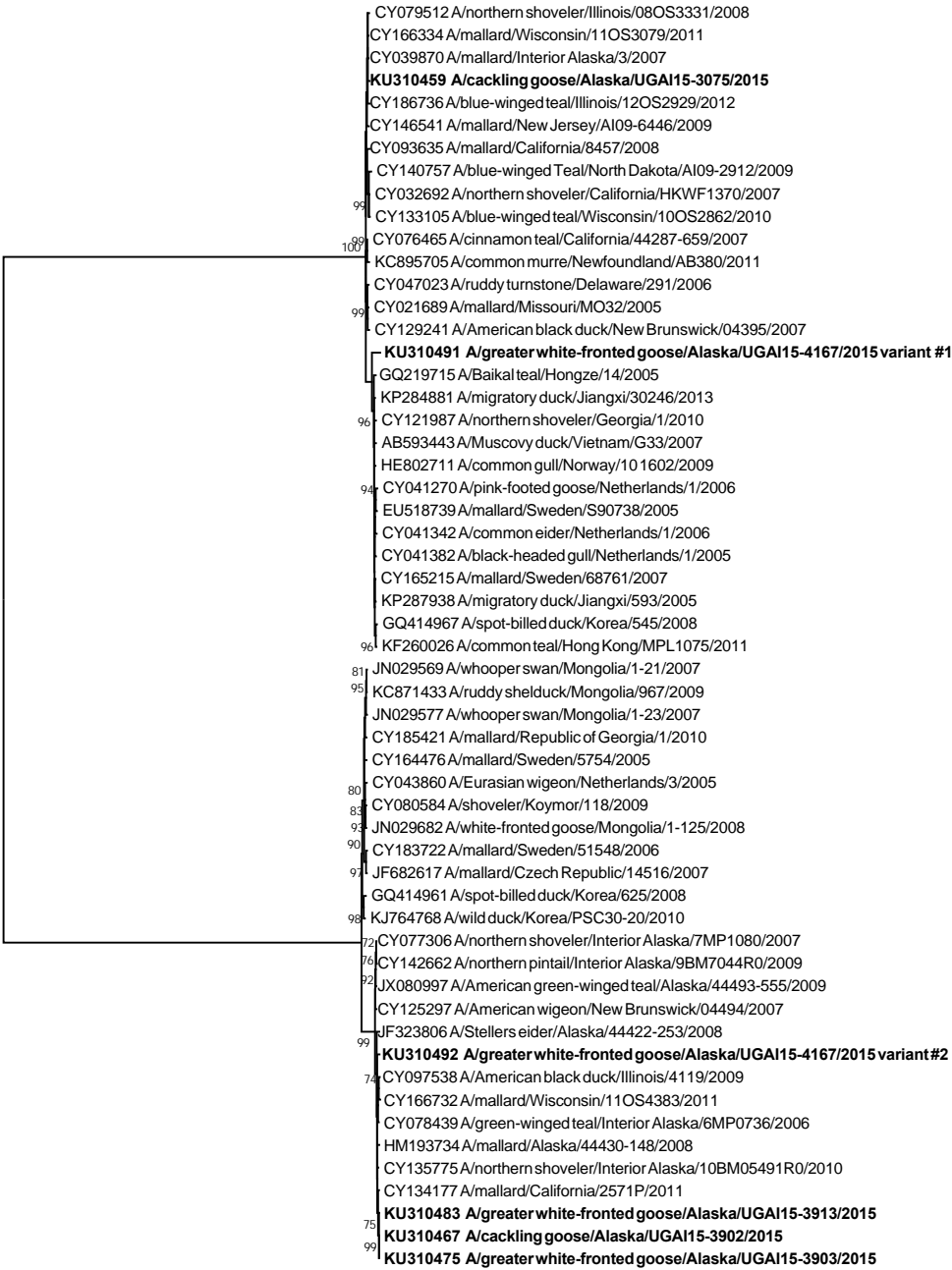

0.5

Supplement: Additional file 2: — Maximum likelihood phylogenies depicting inferred relationship among nucleotide sequences for the PB2 (Figure S1), PB1 (Figure S2), PA (Figure S3), NP (Figure S4), M (Figure S5), and NS (Figure S6) gene segments of influenza A isolates derived from wild geese sampled on the Yukon-Kuskokwim Delta, Alaska during spring 2015 (bold) and others representing continental lineages of influenza A viruses recently detected in North America and Eurasia. Bootstrap support values > 70 for nodes are shown. (PDF 230 kb) [file 12985_2016_511_MOESM2_ESM.pdf]
